# Galectin-3 and Its Relationship With Left Ventricular and Left Atrial Echocardiographic Changes in Hospitalized COVID-19 Patients

**DOI:** 10.7759/cureus.104206

**Published:** 2026-02-24

**Authors:** Nana Egiashvili, Nona Kakauridze, Nino Gvajaia, Luiza Gabunia, Levan Ratiani

**Affiliations:** 1 Department of Internal Medicine, Tbilisi State Medical University, The First University Clinic, Tbilisi, GEO; 2 Department of Clinical Pharmacology, Tbilisi State Medical University, The First University Clinic, Tbilisi, GEO; 3 Department of Intensive Care Medicine, Tbilisi State Medical University, The First University Clinic, Tbilisi, GEO

**Keywords:** cardiac biomarkers, cardiovascular complications, covid-19, echocardiography, galectin-3, left atrial enlargement, left ventricular function, myocardial injury

## Abstract

Galectin-3 has been recognized as a biomarker of inflammation and fibrosis in COVID-19, yet its association with echocardiographic indicators of cardiac involvement remains insufficiently understood. This study aimed to assess the relationship between galectin-3 levels and echocardiographic changes of the left ventricle and left atrium in hospitalized patients with COVID-19. A retrospective analysis was conducted on 41 adults who were divided into moderately symptomatic patients (21/41, 51.2%; within the first week of symptom onset) and severely symptomatic patients (20/41, 48.8%; during the second to third week). All patients underwent standard laboratory testing, including galectin-3 measurement, and transthoracic echocardiography upon admission. Galectin-3 levels were significantly higher in the severe group compared with the moderate group (13.7 ± 7.9 ng/mL vs. 8.7 ± 6.2 ng/mL; p = 0.029). Severe illness was also associated with a larger ascending aortic diameter (p = 0.041) and greater left atrial size (p = 0.05). Although interventricular septal thickness, posterior wall thickness, and left ventricular end-diastolic diameter were similar between groups, left ventricular end-diastolic volume, end-systolic volume, and left ventricular mass were higher in severe cases, but these differences did not reach statistical significance. Left ventricular ejection fraction remained normal in moderate cases and was mildly reduced in severe cases without a statistically significant difference. Galectin-3 demonstrated significant correlations with several echocardiographic markers, most notably left atrial diameter and indices of left ventricular structure and function. These findings suggest that elevated galectin-3 levels are associated with structural myocardial changes in COVID-19. Combined assessment of galectin-3 and echocardiographic parameters may aid in identifying early myocardial involvement and improving cardiovascular risk stratification in hospitalized COVID-19 patients.

## Introduction

Cardiovascular involvement represents one of the major determinants of morbidity and mortality in patients with COVID-19, with both the lungs and the heart frequently affected during the course of the disease [1,2]. The development or worsening of coronary heart disease (CHD) may occur through direct myocardial injury or through secondary mechanisms such as hypoxia and systemic inflammation [3]. Adverse outcomes are particularly common in individuals with pre-existing CHD [4,5], yet new-onset cardiac complications, including acute myocardial infarction, myocarditis, arrhythmias, and acute heart failure, are also observed in patients without known cardiovascular disease (CVD). Approximately 12% of hospitalized patients without prior CHD demonstrate elevated troponin levels or sudden cardiac arrest during infection, indicating significant myocardial involvement [6].

These concerns have intensified interest in identifying biomarkers capable of detecting early cardiac injury in COVID-19. Galectin-3, a β-galactoside-binding lectin involved in inflammation and fibrosis, has gained attention due to its established prognostic relevance in CVD. Findings from the Atherosclerosis Risk in Communities (ARIC) cohort demonstrated that elevated circulating galectin-3 is associated with the development of heart failure, coronary artery disease, ischemic stroke, cardiometabolic dysfunction, and all-cause mortality [7-9]. Much of the foundational evidence for galectin-3 as a fibrosis biomarker comes from pre-COVID-19 cardiovascular cohorts; however, emerging SARS-CoV-2-specific studies suggest galectin-3 also tracks acute disease severity and may reflect early myocardial involvement during infection. Its potential role in predicting COVID-19 severity, fibrotic complications, and cardiovascular outcomes has been highlighted in recent investigations [10].

Echocardiography is an essential tool for assessing cardiac involvement during COVID-19. Guidelines from the European Society of Cardiology recommend focused or point-of-care echocardiography to identify structural and functional abnormalities that may influence clinical management [11]. Clinical studies from China have demonstrated that echocardiographic findings correlate with both direct and indirect cardiac injury in COVID-19 [12]. However, the combined use of galectin-3 and echocardiographic parameters, particularly those related to left ventricular (LV) and left atrial (LA) structure, has not been comprehensively evaluated. In particular, there remains limited evidence on whether combining a fibrosis-related biomarker with routinely obtained echocardiographic structural indices improves early detection of myocardial involvement or refines short-term cardiovascular risk stratification during acute COVID-19. This gap is clinically relevant because echocardiography may identify subclinical structural change even when conventional systolic function parameters remain near normal.

While right ventricular (RV) dysfunction is frequently described in COVID-19, we focused on LV and LA structural parameters because they (i) have well-established prognostic significance across CVDs and (ii) may reflect early increases in left-sided filling pressures and inflammatory-fibrotic signaling during acute infection. RV-focused analyses were beyond the scope of the current report.

## Materials and methods

This retrospective study was conducted at the First University Clinic of Tbilisi State Medical University and included 41 hospitalized adult patients with confirmed COVID-19. All patients were evaluated within 24 hours of admission and monitored throughout their hospital stay. Cardiac complications and troponin elevations were recorded during the index hospitalization. Troponin values reported in this study refer to measurements obtained on admission (within the first 24 hours) as part of routine laboratory evaluation; additional events occurring later during hospitalization were documented descriptively. Patients aged 18 years or older with reverse transcription (RT)-PCR-confirmed SARS-CoV-2 infection using nasopharyngeal or oropharyngeal swabs and with adequate echocardiographic image quality were eligible for inclusion. Exclusion criteria included age under 18 years, pregnancy, dialysis, mechanical ventilation at admission, poor echogenicity, and the presence of type 1 or type 2 diabetes (with the exception of steroid-induced hyperglycemia). Patients with procedure-related myocardial infarction (types 4 or 5) were also excluded. Patients with type 1 or type 2 diabetes were excluded to reduce metabolic confounding, as diabetes is associated with baseline elevations in inflammatory/fibrotic biomarkers and structural cardiac changes that could bias associations between galectin-3 and echocardiographic measures in a small cohort.

Patients were categorized according to the Chinese guideline criteria for COVID-19 severity. Severe disease was defined by the presence of at least one of the following: dyspnea with respiratory rate ≥ 30 breaths/min, resting oxygen saturation ≤ 93%, PaO₂/FiO₂ ≤ 300, or lung infiltrates > 50% within 24-48 hours on chest imaging. Patients not meeting these criteria (mild or moderate pneumonia) were classified as non-severe. In this study, Group 1 (“moderate”) included hospitalized patients with non-severe (moderate pneumonia) presentation during the first week of symptom onset, whereas Group 2 (“severe”) included patients meeting severe criteria during the second to third week of illness [11].

Echocardiographic evaluation followed the criteria from the American Heart Association and the New York Heart Association functional classification system for identifying heart failure [13]. All patients underwent standard transthoracic echocardiography (TTE) performed according to the recommendations of the American Society of Echocardiography [14]. Using two-dimensional imaging, M-mode, linear measurements, and color Doppler, the following parameters were assessed: LV mass, posterior wall thickness, interventricular septal thickness in diastole, and LV end-diastolic diameter, in accordance with established echocardiographic protocols [15]. LA size was assessed using LA diameter because LA volume index (LAVI) was not consistently available in this retrospective cohort, and LA diameter was routinely measured in all included studies. Galectin-3 levels were measured at admission as part of routine laboratory evaluation. Galectin-3 blood sampling and TTE were performed during the same clinical assessment and within 24 hours of hospitalization. Symptom onset date was obtained from the medical records and used to define week-of-illness grouping (week 1 vs. weeks 2-3). Where appropriate, structural measurements were indexed to body surface area (BSA), including LV mass index (LVMI) and LAVI. Measurements were averaged over three consecutive cardiac cycles; inter- and intra-observer variability was assessed in a randomly selected subset and showed acceptable reproducibility.

Statistical analyses were performed using IBM SPSS Statistics (IBM Corp., Armonk, NY, USA). Continuous variables are presented as mean ± standard deviation (SD), and categorical variables as frequencies and percentages. Between-group comparisons were performed using independent-samples t-tests when parametric assumptions were met; normality and homogeneity of variances were assessed, and non-parametric tests were used when assumptions were not met. Associations between galectin-3 and echocardiographic parameters were analyzed using Spearman’s rank correlation coefficient because several variables were not normally distributed and linearity could not be assumed. Binary echocardiographic variables (e.g., valvular regurgitation) were coded as 0/1 and analyzed using Spearman’s rank correlation as a rank-based measure of association; results were interpreted cautiously given the binary nature of these variables. A two-sided p-value < 0.05 was considered statistically significant. Given the exploratory nature of analyses and the limited sample size, p-values were not adjusted for multiple comparisons, and results were interpreted cautiously. No missing data were present for variables included in the final analyses. Normality and homogeneity of variances were assessed for each continuous echocardiographic variable prior to applying independent-samples t-tests. Because several structural echocardiographic measures are interrelated (collinearity), correlation findings may reflect shared underlying remodeling; therefore, individual correlations should not be interpreted as independent effects.

The research protocol was reviewed and approved by the Tbilisi State Medical University Research Ethics Committee (Meeting N4, Approval No. 2022/97; review dates: July 22, 2022-August 2, 2022). The requirement for informed consent was waived due to the retrospective nature of the study.

## Results

Patients were classified into the moderate (non-severe) and severe groups according to the Chinese guideline criteria described in the Materials & Methods. The mean age was 72.2 ± 11.7 years in the moderate group and 77.1 ± 10.2 years in the severe group, with no statistically significant difference. Body mass index (BMI) was 26.1 ± 4.4 kg/m² in the moderate group and 28.7 ± 5.0 kg/m² in the severe group. Systolic and diastolic blood pressure did not differ significantly between groups. Resting oxygen saturation was significantly lower among severe cases (89.4 ± 1.5%) compared with moderate cases (93.4 ± 1.4%; p < 0.001). Baseline clinical characteristics are summarized in Table 1. BMI was numerically higher in the severe group, but the difference did not reach statistical significance. There were no statistically significant differences between the two groups in age, BMI, or systolic and diastolic blood pressure (Table 1). Ischemic heart disease was present in 28.6% of patients in Group 1 and 45% in Group 2, while hypertension occurred in 76% of Group 1 and 90% of Group 2 (Table 2). These between-group differences in comorbidity prevalence should be considered when interpreting the observed associations. Creatinine differed significantly between groups (p = 0.043), and glucose showed a borderline difference (p = 0.050) (Table 1).

**Table 1:** Clinical characteristics by group Values are presented as mean (SD). Group comparisons were performed using an independent-samples t-test. BMI: body mass index; BP: blood pressure; SD: standard deviation.

Variable	Group 1 (moderate), n = 21, mean (SD)	Group 2 (severe), n = 20, mean (SD)	t	p-value
Age (years)	72.2 (11.7)	77.7 (10.2)	1.34	0.188
BMI (kg/m²)	26.1 (4.4)	28.7 (5.0)	1.77	0.085
Systolic BP (mmHg)	133.9 (20.2)	137.1 (14.6)	0.579	0.566
Diastolic BP (mmHg)	79.2 (12.7)	78.4 (11.1)	0.214	0.831
Galectin-3 (ng/mL)	8.7 (6.2)	13.7 (7.9)	2.261	0.029
Oxygen saturation at rest (%)	93.4 (1.4)	89.4 (1.5)	8.832	<0.001

**Table 2:** Cardiovascular risk factors by group Data are presented as n/N (%). p-values were obtained using Pearson’s chi-squared (χ²) test (two-sided). Degrees of freedom (df) and effect size (φ) are reported for each comparison; for 2 × 2 tables, φ is equivalent to Cramer’s V. CVD: cardiovascular disease.

Risk factor	Group 1 (moderate), n = 21	Group 2 (severe), n = 20	χ² (df), N	p-value	Effect size (φ)
Arterial hypertension	16/21 (76.2%)	18/20 (90.0%)	1.38 (1), 41	0.24	0.18
Obesity	3/21 (14.3%)	7/20 (35.0%)	2.38 (1), 41	0.123	0.24
Smoking	10/21 (47.6%)	10/20 (50.0%)	0.02 (1), 41	0.879	0.02
Coronary heart disease (CHD)	6/21 (28.6%)	9/20 (45.0%)	1.19 (1), 41	0.275	0.17
Family history of CVD	5/21 (23.8%)	10/20 (50.0%)	3.03 (1), 41	0.082	0.27

Cardiovascular risk factors were similarly distributed between groups (Table 2). Arterial hypertension was the most prevalent comorbidity (16/21, 76.2% vs. 18/20, 90.0%) and did not differ significantly between moderate and severe cases (χ²(1, N = 41) = 1.38, p = 0.240, φ = 0.18). CHD (6/21, 28.6% vs. 9/20, 45.0%; χ²(1, N = 41) = 1.19, p = 0.275, φ = 0.17) and family history of CVD (5/21, 23.8% vs. 10/20, 50.0%; χ²(1, N = 41) = 3.03, p = 0.082, φ = 0.27) were more frequent in the severe group, but these differences were not statistically significant. Likewise, no significant between-group differences were observed for obesity (3/21, 14.3% vs. 7/20, 35.0%; χ²(1, N = 41) = 2.38, p = 0.123, φ = 0.24) or smoking history (10/21, 47.6% vs. 10/20, 50.0%; χ²(1, N = 41) = 0.02, p = 0.879, φ = 0.02). Overall, effect sizes ranged from negligible to small-to-moderate (φ = 0.02-0.27).

**Figure 1:**
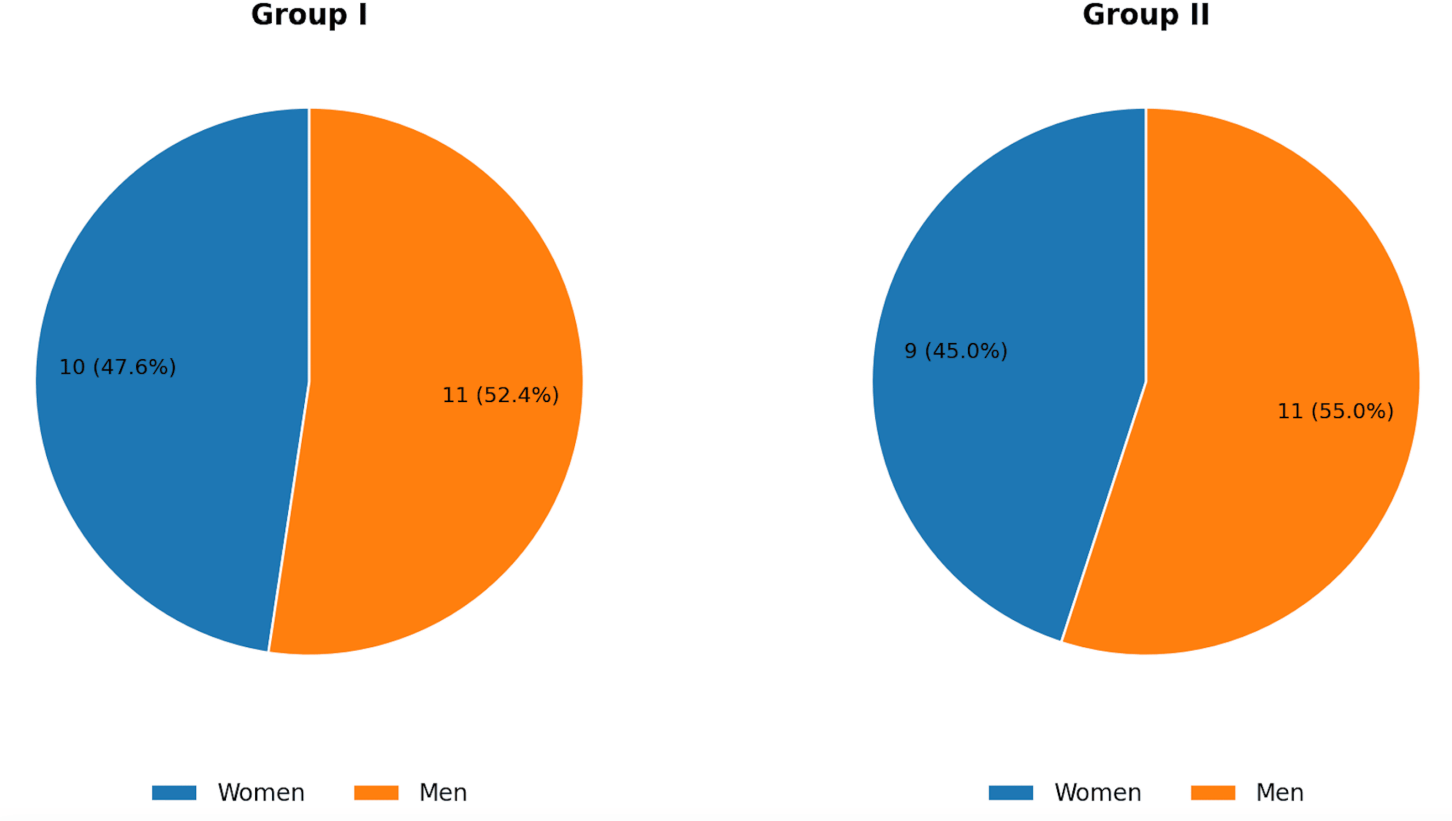
Sex distribution in Groups I and II

Sex distribution is presented in Figure 1. Sex distribution did not differ between groups (χ² = 0.027, p = 0.87).

Galectin-3 levels were significantly higher in the severe group (13.7 ± 7.9 ng/mL) compared with the moderate group (8.7 ± 6.2 ng/mL; p = 0.029). This suggests more pronounced inflammatory and fibrotic activity in severe illness.

Aortic evaluation showed that the aortic root diameter did not differ significantly between groups (3.43 ± 0.23 cm vs. 3.50 ± 0.39 cm; p = 0.486). However, the ascending aorta diameter was significantly greater in the severe group (3.68 ± 0.35 cm) compared with the moderate group (3.48 ± 0.25 cm; p = 0.041).

No statistically significant differences were observed in interventricular septal thickness or posterior wall thickness (p = 0.456 and p = 0.526, respectively). LV end-diastolic diameter was within the normal range for both groups (p = 0.330). LV end-diastolic volume, end-systolic volume, and LV mass were all higher in severe cases, approaching statistical significance. LV ejection fraction was normal in the moderate group (52.5 ± 7.1%) and mildly reduced in severe patients (49.7 ± 6.1%), though the difference was not statistically significant (p = 0.184). Fractional shortening, cardiac index, stroke volume, cardiac output, and E/e′ ratio were also similar across groups. Detailed echocardiographic measurements are shown in Table 3. Because hypertension and ischemic heart disease were more prevalent in the severe group, structural differences should be interpreted cautiously, as baseline comorbidities may contribute.

**Table 3:** Echocardiographic data by group Values are presented as mean (SD). Between-group comparisons were performed using the independent-samples t-test. Ao: aortic root diameter; Asc Ao: ascending aorta diameter; IVS: interventricular septal thickness; PW: posterior wall thickness; LVEDD: left ventricular end-diastolic diameter; LVEDV/LVESV: left ventricular end-diastolic/systolic volume; LV mass: left ventricular mass; LA: left atrial diameter; EF: ejection fraction; FS: fractional shortening; CI: cardiac index; SV: stroke volume; CO: cardiac output; E/e′: mitral inflow velocity to annular velocity ratio; SD: standard deviation. LVEDV, LVESV, and LV mass are reported as absolute values (not indexed to the body surface area).

Variable	Group 1 (moderate), n = 21, mean (SD)	Group 2 (severe), n = 20, mean (SD)	t	p-value
Ao (cm)	3.43 (0.23)	3.50 (0.39)	0.704	0.486
Asc Ao (cm)	3.48 (0.25)	3.68 (0.35)	2.114	0.041
IVS (cm)	1.29 (0.17)	1.33 (0.17)	0.753	0.456
PW (cm)	1.17 (0.14)	1.20 (0.16)	0.64	0.526
LVEDD (mm)	49.4 (3.7)	50.7 (4.7)	0.987	0.33
LVEDV (mL)	112.2 (13.0)	122.0 (22.9)	1.696	0.098
LVESV (mL)	53.8 (12.5)	62.2 (18.7)	1.699	0.097
LV mass (g)	81.0 (24.4)	98.6 (36.4)	1.827	0.075
LA (cm)	3.9 (0.4)	4.1 (0.5)	1.969	0.056
EF (%)	52.5 (7.1)	49.7 (6.1)	1.351	0.184
FS (%)	26.2 (3.5)	25.0 (2.9)	1.192	0.24
CI (L/min/m²)	2.46 (0.62)	2.63 (0.49)	0.971	0.338
SV (mL)	58.5 (7.4)	59.8 (7.8)	0.548	0.587
CO (L/min)	4.53 (1.18)	5.11 (1.15)	1.593	0.119
E/e′ (ratio)	9.0 (2.6)	9.1 (3.2)	0.11	0.913

LA diameter was significantly larger in the severe group (4.1 ± 0.5 cm) compared with the moderate group (3.9 ± 0.4 cm; p = 0.05), indicating early atrial remodeling associated with increased disease severity. Correlation analysis revealed strong associations between galectin-3 and multiple echocardiographic parameters. Galectin-3 showed a strong positive correlation with LA size in both groups (Group 1: r = 0.7369, p = 0.000; Group 2: r = 0.7024, p = 0.001). Positive correlations were also observed with interventricular septal thickness, posterior wall thickness, and LV mass. Inverse correlations were found with LV ejection fraction (Group 1: r = -0.5622, p = 0.008; Group 2: r = -0.5142, p = 0.020), fractional shortening, and stroke volume (significant in Group 1). Correlation coefficients are presented in Table 4.

**Table 4:** Spearman correlations between galectin-3 and echocardiographic parameters Values are Spearman’s rank correlation coefficients (ρ). Mitral regurgitation and aortic regurgitation were coded as binary variables (present/absent). Ao: aortic root diameter; Asc Ao: ascending aorta diameter; IVS: interventricular septal thickness; PW: posterior wall thickness; LVEDD: left ventricular end-diastolic diameter; LVEDV/LVESV: left ventricular end-diastolic/systolic volume; LV mass: left ventricular mass; LA: left atrial diameter; EF: ejection fraction; FS: fractional shortening; CI: cardiac index; SV: stroke volume; CO: cardiac output.

Parameter	ρ (Group 1, n = 21)	ρ (Group 2, n = 20)	p (Group 1)	p (Group 2)
Ao (cm)	0.378	-0.087	0.091	0.715
Asc Ao (cm)	0.4283	0.0889	0.053	0.709
IVS (cm)	0.5419	0.4335	0.011	0.056
PW (cm)	0.5477	0.4199	0.01	0.065
LVEDD (mm)	0.2852	0.1435	0.21	0.546
LVEDV (mL)	0.166	0.1373	0.472	0.564
LVESV (mL)	0.4395	0.293	0.046	0.21
LV mass (g)	0.3665	0.2515	0.102	0.285
LA (cm)	0.7369	0.7024	<0.001	0.001
EF (%)	-0.5622	-0.5142	0.008	0.02
FS (%)	-0.4964	-0.4976	0.022	0.026
CI (L/min/m²)	-0.139	-0.3563	0.548	0.123
SV (mL)	-0.4548	-0.3003	0.038	0.198
CO (L/min)	-0.031	-0.2719	0.894	0.246
Mitral regurgitation (present vs. absent)	0.4017	0.6402	0.071	0.002
Aortic regurgitation (present vs. absent)	0.2884	0.441	0.205	0.052
E/A (ratio)	0.4315	0.2997	0.051	0.199

## Discussion

This study demonstrates that circulating galectin-3 levels and several echocardiographic parameters of LV and LA structure reflect the degree of myocardial involvement in hospitalized COVID-19 patients. The significantly higher galectin-3 concentrations observed in the severe group (13.7 ± 7.9 vs. 8.7 ± 6.2 ng/mL; p = 0.029) support existing evidence indicating its role as a biomarker of inflammatory and fibrotic activity in cardiac disease [16-18]. Recent studies have also suggested that galectin-3 may serve as an indicator of COVID-19 severity and tissue remodeling, reinforcing the findings of our analysis [19].

Galectin-3 is implicated in macrophage activation, cytokine amplification, and fibroblast proliferation, pathways that can promote myocardial inflammation and extracellular matrix remodeling. In acute COVID-19, systemic inflammation, endothelial dysfunction, and microvascular injury may increase myocardial stress and impair diastolic relaxation, which can elevate LV filling pressures and lead to LA dilation. These processes may provide a biologic rationale for the observed associations between higher galectin-3 levels and echocardiographic indicators of LA/LV structural change early in hospitalization. Galectin-3 has been broadly studied as a biomarker of inflammation and fibrosis in CVD; therefore, its association with cardiac structure on echocardiography is biologically plausible, although COVID-19-specific data remain limited.

A notable observation in this cohort was the enlargement of the ascending aorta in severely symptomatic patients. Although the aortic root diameter was similar between groups, the ascending aorta was significantly wider in severe cases (3.68 ± 0.35 vs. 3.48 ± 0.25 cm; p = 0.041). This finding aligns with reports proposing that COVID-19 can cause vascular inflammation, endothelial dysfunction, and immune-mediated injury to large vessels [20]. In some patients, latent vascular abnormalities may become clinically evident during acute infection, which may explain the variability in aortic dimensions observed across disease severity [20].

The finding of ascending aortic enlargement should be interpreted cautiously because it may represent pre-existing or incidental pathology rather than a COVID-19-specific effect in the absence of baseline imaging. At present, the prognostic relevance of mild aortic enlargement in acute COVID-19 remains uncertain in this dataset.

Our findings demonstrate associations rather than causation. Galectin-3 should be interpreted primarily as a biomarker reflecting inflammatory-fibrotic activity and possible myocardial stress during acute COVID-19, rather than a proven direct mediator of the observed echocardiographic changes.

Interventricular septal thickness, posterior LV wall thickness, and LV end-diastolic diameter did not differ significantly between groups, suggesting that concentric LV remodeling may not be a prominent early feature of non-critical COVID-19. However, LV end-diastolic volume, LV end-systolic volume, and LV mass were consistently higher in the severe group and approached statistical significance. These parameters have been shown to predict adverse outcomes and mortality in CVD, including in large trials such as MADIT-CRT (Multicenter Automatic Defibrillator Implantation Trial With Cardiac Resynchronization Therapy) [21]. The trends observed in our study suggest that LV dilation and increased LV mass may indicate early myocardial stress or impaired ventricular function in COVID-19.

LV ejection fraction remained within the normal range in the moderate group, whereas mildly reduced values were observed in severe cases, although the difference was not statistically significant. This pattern is consistent with earlier studies reporting subclinical systolic dysfunction in COVID-19, even in the absence of overt heart failure [12]. Functional indices such as fractional shortening, cardiac index, stroke volume, cardiac output, and the E/e′ ratio also showed no significant differences, indicating that traditional echocardiographic markers of global function may not fully reflect disease progression in this population.

In contrast, LA diameter demonstrated a statistically significant increase (4.1 ± 0.5 vs. 3.9 ± 0.4 cm; p = 0.05) in patients with severe disease. LA enlargement is a well-established marker of impaired diastolic filling, elevated left-sided pressures, atrial remodeling, arrhythmia risk, stroke, and heart failure [22]. The significant correlation between galectin-3 and LA size observed in both groups highlights the link between inflammatory-fibrotic activation and atrial structural changes during COVID-19.

Correlation analyses further demonstrated that higher galectin-3 levels were associated with increased interventricular septal thickness, posterior wall thickness, and LV mass, as well as reduced LV ejection fraction and fractional shortening. These findings suggest that galectin-3 may help identify subtle myocardial dysfunction and structural remodeling before overt systolic impairment becomes pronounced. Together, these results indicate that combining galectin-3 assessment with echocardiographic evaluation may enhance early detection of cardiac involvement and improve risk stratification in hospitalized patients with COVID-19.

The echocardiographic findings were obtained early during hospitalization and may reflect acute, potentially transient effects of systemic inflammation (e.g., altered loading conditions, myocardial edema, and increased stiffness) rather than established chronic remodeling alone. However, acute inflammatory-fibrotic signaling may also represent an early phase of longer-term remodeling. Longitudinal follow-up imaging would be required to distinguish transient changes from persistent structural remodeling. Because age, hypertension, and CHD can influence both galectin-3 levels and cardiac structure, residual confounding cannot be excluded when interpreting group differences and correlations in this cohort.

Regarding baseline comorbidities, traditional cardiovascular risk factors were similarly distributed between the moderate and severe groups (Table 2). Although CHD and a family history of CVD were more frequent among patients with severe disease, these differences were not statistically significant (CHD: χ²(1, N = 41) = 1.19, p = 0.275, φ = 0.17; family history of CVD: χ²(1, N = 41) = 3.03, p = 0.082, φ = 0.27). The lack of statistical significance may reflect the modest sample size (N = 41), which limits power to detect small between-group differences. Notably, arterial hypertension was highly prevalent in both groups (>75%) (χ²(1, N = 41) = 1.38, p = 0.240, φ = 0.18), highlighting a substantial burden of underlying cardiovascular pathology in this hospitalized cohort, independent of acute disease severity.

If validated, combining galectin-3 with admission echocardiography could help identify patients who warrant closer cardiovascular monitoring (e.g., repeat echocardiography, stricter fluid/volume management, and lower threshold for cardiology consultation), particularly when structural changes are present despite preserved systolic function. This integrated approach may support risk stratification for short-term decompensation and targeted follow-up after discharge.

In this study, the proposed utility of integrating galectin-3 with echocardiography is intended primarily for early inpatient screening/risk stratification and monitoring of patients at a higher likelihood of cardiac involvement during hospitalization. Prognostic use for long-term outcomes requires outcome-based validation in larger cohorts.

This study has several limitations inherent to its single-center, retrospective design. The modest sample size (N = 41) may restrict statistical power and the generalizability of our findings to broader populations. Since patients were stratified by symptom onset and clinical severity, observed differences might reflect the natural progression of the disease rather than severity alone. Furthermore, because galectin-3 levels and echocardiographic parameters were assessed only at admission without pre-infection baselines, we cannot definitively establish causality or track longitudinal changes. Future prospective studies with larger cohorts and serial assessments are warranted to validate these associations. Because this was a retrospective study, echocardiographers were not formally blinded to clinical severity or galectin-3 results, and measurement bias cannot be excluded. Exclusion of patients with diabetes may limit generalizability, as diabetes is common among hospitalized COVID-19 populations. Confidence intervals for between-group differences were not routinely reported, and this limits the precision of estimates. Although age and BMI were not statistically different between groups, we did not adjust for these variables due to the small sample size and risk of model overfitting. Although BMI did not differ significantly between groups, residual confounding by BMI cannot be fully excluded given the small sample size. Treatment heterogeneity (e.g., corticosteroids, antivirals, and supportive therapies) may influence inflammatory biomarkers and hemodynamics and could therefore affect galectin-3 levels and echocardiographic findings; medication-level adjustment was not feasible in this cohort.

We did not include RV function or pulmonary pressure parameters, which limits the assessment of global and pulmonary vascular cardiac involvement commonly described in COVID-19. Future studies should include (i) longitudinal echocardiographic follow-up to determine whether observed structural changes are transient or persist, (ii) outcome-based validation to assess whether galectin-3 adds prognostic value for clinical endpoints, and (iii) comparisons with established cardiac biomarkers (e.g., troponin and natriuretic peptides) and multimarker strategies.

## Conclusions

This study demonstrates that elevated galectin-3 levels, together with specific echocardiographic changes of the LV and LA, reflect the degree of myocardial involvement in hospitalized COVID-19 patients. Higher galectin-3 concentrations were associated with markers of structural cardiac remodeling, including increased LA size, greater LV volumes, and mildly reduced systolic function in more severe disease. These findings suggest that the combined assessment of galectin-3 and echocardiographic parameters may help identify early cardiac injury, stratify risk, and guide clinical evaluation in patients with COVID-19. Further studies with larger cohorts are needed to confirm these associations and clarify their prognostic implications.
